# Feasibility of Using College Students to Increase Cancer Screening Behaviors Among Their Parents

**DOI:** 10.3390/ijerph23020246

**Published:** 2026-02-16

**Authors:** Caitlin C. Abar, Logan Robert Kayser, Amanda Lewis, Hannah Randolph, Beau Abar

**Affiliations:** 1 Department of Psychology, Philosophy, and Neuroscience, SUNY Brockport, Brockport, NY 14420, USA; 2 Department of Psychology, SUNY Brockport, Brockport, NY 14420, USA; lkays1@brockport.edu (L.R.K.); alewi8@brockport.edu (A.L.); hrand2@brockport.edu (H.R.); 3 Departments of Emergency Medicine, Psychiatry, and Public Health Sciences, University of Rochester Medical Center, Rochester, NY 14642, USA; beau_abar@urmc.rochester.edu

**Keywords:** cancer screening, parents, intervention, feasibility, college students

## Abstract

**Highlights:**

**Public health relevance—How does this work relate to a public health issue?**

- Interval cancer screening represents one of the most powerful methods we have for mitigating cancer-related morbidity and mortality.
- A substantial proportion of individuals eligible for screening and not up-to-date with recommended screenings.

**Public health significance—Why is this work of significance to public health?**

- New methods of intervention are needed to reach the segment of eligible individuals who are not up-to-date with screenings.
- Family members represent an available and important resource that has largely been unused in efforts to increase cancer screening.

**Public health implications—What are the key implications or messages for practitioners, policy makers, and/or researchers in public health?**

- College students are highly willing to talk with parents and other adults about cancer screening if the needed information is provided to them.
- Intervening with college students using online materials is feasible and potentially effective at reaching unscreened parents and other close adults.

**Abstract:**

This study evaluated the feasibility of using college students to encourage cancer screening among their parents or other close adults. Methods: A sample of college students were surveyed on their willingness to discuss cancer screening with their parents and their perceived importance of cancer screening. Individuals reporting high levels (≥7/10) on each were offered a brief intervention (i.e., basic cancer screening information) and a follow-up survey. Results: A total of 189 students completed the baseline survey. A subset of 92 students met intervention criteria (49%), with 54 of 92 accepting (59%). Of those who accepted, 19 of 54 were reached at follow-up (35%). Roughly half of those reached engaged in cancer screening discussions with a parent, most speaking with their mother. Open-ended feedback was positive and commonly focused on the desire for additional knowledge. Conclusions: Results demonstrate that college students are a promising target for future intergenerational intervention work.

## 1. Introduction

Timely preventive cancer screening efforts have been responsible for a substantial portion of the lives saved over the past several decades among individuals with breast, cervical, colorectal, lung, and prostate cancers [1,2]. The United States Preventive Services Task Force (USPSTF) issued Grade A or B recommendations for interval breast, cervical, colorectal, and lung cancers, indicating the existing evidence demonstrates at least a moderate net health benefit from adherence with screening guidelines [3,4,5,6]. Federal legislation in the U.S. ensures that these screenings are covered by private insurance and Medicaid with minimal or no out-of-pocket costs to patients [7,8,9,10]. Furthermore, many healthcare organizations offer screening services to individuals experiencing barriers to screening [11,12,13,14,15].

Many adults, even those with a primary care provider (PCP), remain non-adherent with recommended cancer screenings [16,17,18]. Recent reports from the U.S. Centers for Disease Control and Prevention indicate that 20% of eligible women are not up-to-date with breast cancer screening; 25% of eligible women are not up-to-date with cervical cancer screening;, and 33% of men and women are not up-to-date with colorectal cancer screening [19]. Estimates from the U.S. Behavioral Risk Factor Surveillance System show that approximately 18% of eligible adults are up-to-date with lung cancer screening [20]. Rates of screening have shown to be even lower among ethnic and racial minorities and individuals with lower socioeconomic status [19,20]. These estimates are suboptimal, causing broad initiatives such as Healthy People 2030 to call for increased screening rates [21,22,23]. Consequently, new methods encouraging screening in adult populations are needed to achieve increased cancer screenings. Interventions making use of family members to encourage screening represent an understudied and potentially valuable area of inquiry. Family members, as agents of health-related change, possess several advantages that can be leveraged. First, family members have ready access to adults in need of cancer screening intervention. This access cannot be overstated, as screening rates are troubling among those with PCPs, but far lower among those without a PCP who are disconnected from the healthcare system. These individuals are not reachable through standard, clinic-based screening interventions. Second, family members represent trusted sources of health information [24,25], behind only health care providers. Third, theoretical frameworks [26,27,28] supported by empirical results have demonstrated the role family can play in establishing and sustaining health behaviors [29]. Specifically, the Health-Promoting Family Model highlights the proximal roles that family health values and history of health practices influence current family health practices, while operationalizing children in the family as health-promoting actors [26]. Similarly, Family Systems Theory details the bidirectional roles parents and children play with respect to health outcomes, with parents influencing children and children influencing parents [27]. Finally, and most germane to the current work, previous acceptability research has demonstrated that family members, recruited from either college campuses or Emergency Departments, are highly willing to talk with family members about cancer screening, particularly if screening-related information is provided to them prior [30,31].

### Study Objectives

The current study seeks to build upon previous acceptability work by determining the feasibility of implementing an intergenerational intervention using college students to catalyze cancer screening behaviors among their parents and/or grandparents. College students are particularly open to health information and intervention [32,33,34] and generally report high-quality [35], engaged [36] relationships with parents/family members. In this study, we aimed to (a) demonstrate methods for identifying potential high-yield intervention participants among college students, (b) pilot the implementation of a brief, information-based intervention online with willing participants, and (c) follow up with students to evaluate outcomes and obtain formative feedback. The intervention was low-intensity (i.e., a single link to a screening information website), such that the focus was on operational feasibility and lessons learned in preparation for a formal intervention trial.

## 2. Materials and Methods

### 2.1. Participants

College students at SUNY Brockport were recruited from Introduction to Psychology (PSH 110) sections using SONA, a resource used to link students with research participation opportunities. Students in PSH 110 are required to participate in several research studies to receive credit. Completion of our baseline survey satisfied course requirements. Students between the ages of 18 and 23 were eligible to participate as they represented the typical college-student age range and did not require the inclusion of parents in the consent procedures, which might have biased results.

### 2.2. Measures

#### 2.2.1. Baseline

**Family Members.** Participants responded about whether they had at least one (a) mother figure in their lives and (b) whether they had at least one father figure in their lives.

**Willingness to Discuss Cancer Screening.** Participants responded to the general prompt, “Rate on a scale from 1 to 10, with 1 meaning not willing at all and 10 meaning completely willing, the extent to which you would be willing to talk with your parents or other close adults about receiving preventive cancer screenings.” They then denoted who they would be willing to speak with (mother, father, grandparent(s), others). Participants also reported which screenings they would be willing to discuss. Participants who reported a willingness of ≤2 were asked the open-ended question, “What makes you unwilling to discuss cancer screenings with parents or other close adults? If you prefer not to answer, please type ‘NC’.” Participants who reported a willingness of >2 and <10 were asked the open-ended question, “What might make you more willing to speak with parents or other close adults about cancer screening? For example, are there specific resources you would want access to?” Participants who scored 10 were not asked this question, as they had already indicated complete willingness. All participants were asked about the expected discussion outcomes, “If you spoke with your parents or other close adults about cancer screening, how much of an impact do you think you would have? (My input would have no effect on them getting screened; They might decide to get screened; They would very likely get screened; They would absolutely get screened).”

**Importance of Screening and Intentions to Receive Screening.** Participants were prompted with a brief description of United States Preventive Services Task Force recommended screenings and eligibility (“The United States Preventive Services Task Force recommends adults receive 4 different types of cancer screening as a way to limit cancer-related deaths. Individuals with cervixes are recommended to receive cervical cancer screening between the ages of 21 and 65. Women aged 50 to 74 are recommended to receive breast cancer screening every other year. Colorectal cancer screening is recommended for everyone between 45 and 75 years of age. Lung cancer screening is recommended for current smokers or those who quit within the past 15 years each year between 50 and 80 years of age.”). Importance of screening was then indexed by their response to the following question: “Rate on a scale from 1 to 10, with 1 meaning not at all important and 10 meaning extremely important, how important receiving recommended preventive cancer screenings is to you.” Intention to screen was then indexed by the following request: “Rate on a scale from 1 to 10, with 1 meaning no intention at all and 10 meaning 100% intent, the extent to which you intend to receive preventive cancer screenings when you become eligible.”

**Covariates.** Participants were asked whether they believe the family members they would talk with currently receive recommended cancer screenings (No, Probably, Definitely, Do not Know). They also answered whether their parents have a primary care provider (“meaning a doctor they see for check-ups and when ill”; asked individually for mothers and fathers). Participants also responded about whether or not they had a primary care provider of their own (yes/no; if yes, was it a pediatrician, a doctor who primarily treats adults, a provider at the campus health center, or other).

Participants responded about relationship quality with their mother and father using 4 questions each on a 5-point scale from strongly disagree to strongly agree (Maternal relationship quality α = 0.90; Paternal relationship quality α = 0.89). Mean scores were created for each parent.

#### 2.2.2. Follow-Up

**Discussions with Family Members.** Engagement in discussions was represented by response to the following: “During the initial study, your responses indicated that you were willing to talk with your parents and/or other close adults about preventive cancer screenings. Have you talked with your parents or any other close adults about cancer screenings since you were first surveyed?” Participants were asked who they spoke with about cancer screening (Mother, Father, Grandparents, Other Relatives, Non-relatives). They then responded about which cancer screenings they discussed (Breast, Cervical, Colorectal, Lung). Participants then answered a series of open-ended questions about the conversations, asking them (a) to describe the content of the discussions and how they felt during the discussions, (b) how their family members seemed to respond, and (c) whether they thought their family members ultimately received screening. For those who thought their family members received screening, participants were asked what made them think this and how sure they were. They finished by answering the questions, “How do you think that the conversations could have gone better? What resources or knowledge do you feel you might have benefited from?”

Among participants who did not engage in cancer screening discussions with family members, we asked why not (I forgot about it; I was too busy with other things; There was never the right time; My parents/other adults were too busy; My parents/other adults were dealing with other serious issues; It did not feel important; Other reasons. Participants could check all that applied.). These individuals were then asked if there was anything that would have made them talk with family members about preventive cancer screening (yes/no) and what that might have been (open-ended).

### 2.3. Procedure

Participant recruitment occurred over an 8-week period during Fall 2024. Interested students were provided with an online information sheet describing the study, followed by a digital informed consent form. Participants then completed a 20–25 min baseline survey on health-related behaviors using Qualtrics [37]. This baseline survey included questions on willingness to speak with family members about cancer and personal intent to screen for cancers when eligible. Individuals who reported a willingness score of 8 or greater and an intention score of 8 or greater were provided a link to the Center for Disease Control and Prevention on USPSTF-recommended cancer screening tests (https://www.cdc.gov/cancer/prevention/screening.html, accessed on 15 August 2024). These individuals were also offered the opportunity to complete a follow-up Qualtrics survey roughly 90 days post-baseline on any cancer screening-related discussions that the participant engaged in with their family members. These thresholds were chosen to maximize efficiency and resource utilization of this pilot study (i.e., only enroll and follow-up with those most likely to engage).

Notably, the intervention was not developed to maximize efficacy; it was instead used to test our method of dissemination (e.g., brief online information provision) for subsequent, refined intervention materials informed by results of the current study. Participants who completed the follow-up were provided a $10 e-gift card.

All study activities were approved by the SUNY Brockport Research Subjects Review Board.

### 2.4. Plan of Analysis

Measures of willingness to speak with family members about cancer screenings, perceived importance of screening, and intentions to screen when eligible were examined using standard descriptive statistics (frequencies and percentages; Means, Standard Deviations, and Inter-Quartile Ranges). Willingness was compared to a previously presented estimate from an independently collected cohort of students from SUNY Brockport [30,31] (Sagastume et al., 2026; Lewis et al., 2025) using a one-sample *t*-test. We anticipated similar levels across cohorts.

Among participants deemed eligible based on baseline responses, we then evaluated rates of (a) acceptance of offer for intervention and follow-up, (b) retention at follow-up (i.e., completion of follow-up survey), and (c) engagement in cancer screening discussions with family members. These discussions were then examined for (a) who was talked to, (b) what was discussed, (c) whether the participant believed their family member got screened, and (d) where this perception came from. All categorical variables were presented using frequencies and percentages. Open-ended responses to questions on facilitators or barriers to conversations and content of subsequent discussions were examined in a quality-improvement framework, such that common and idiosyncratic responses were used to inform subsequent methods and intervention development/refinement. Willingness, intention, and follow-up variables were then related to baseline covariates using Spearman correlations. We performed all analyses using IBM SPSS 28.

## 3. Results

### 3.1. Sample Characteristics

A total of 189 students completed the Qualtrics survey. Participant demographics are summarized in Table 1. The mean age was roughly 19, and the majority of participants were women. Nearly three in four participants self-identified as White, and more than half were in their first year of college. A total of 158 participants reported at least one mother and one father figure (84%), while only four participants reported a lack of either a mother or father figure (2%).

### 3.2. Willingness to Speak with Family Members About Cancer Screening

The average willingness to speak with parents/grandparents/other adults about cancer screening was 7.87 (SD = 2.32; IQR = 7.0–10). A score of 10/10 was reported by 67 students (35%). The current mean value was identical to a previously presented cohort of SUNY Brockport college students (M = 7.87, *p* = 0.983), implying sound independent replication. Most participants were willing to talk with their primary mother figure (*n* = 162, 86%); half were willing to talk with their primary father figure (*n* = 95, 50%); roughly one in four were willing to talk with their grandparents (*n* = 53, 28%), and 15 students (8%) were willing to talk with other adults. A total of 39 students were willing to speak with their mothers, fathers, and grandparents (21%), while 91 reported willingness to speak with at least two family members (48%).

The majority of participants reported willingness to speak with family members about breast cancer screening (*n* = 107, 57%); roughly a quarter of participants would discuss lung cancer (*n* = 49, 26%), and 8–9% would discuss cervical or colorectal cancer (*n* = 16, 15, respectively). Participants’ expectations of the impact of discussions with family members were mixed, with 17 (9%) expecting their input would have no impact on screening, 96 (51%) expecting their family members might get screened, 59 (31%) reporting their family members would very likely get screened, and 16 (9%) expecting their family members would absolutely get screened.

Student perceptions of the importance of cancer screening and intentions to receive cancer screening when eligible were each relatively high (importance M = 7.92, SD = 2.11, IQR = 7.00–10.00; intentions M = 7.74, SD = 2.41, IQR = 7.00–10.00). Willingness to speak with family members, perceived importance of screening, and intentions to screen when eligible were all closely correlated (*r*’s > 0.71, *p*’s < 0.001).

### 3.3. Eligibility and Acceptance of Intervention/Follow-Up

**Figure 1:**
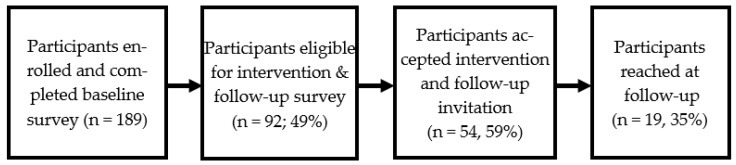
Participant Flow Diagram.

A total of 92 of the 189 participants (49%) met our conservative criteria for intervention/follow-up participation (See Figure 1). Of these, 54 of the 92 eligible individuals received the cancer screening information link and accepted an invitation to be followed up with to discuss subsequent screening discussions (59%). Acceptance was not related to rating of willingness, importance, or intentions (*p*’s > 0.75), at least partly due to restriction of range.

### 3.4. Follow-Up Completion and Outcomes

Nineteen students completed the follow-up survey (35% of the 54 students who accepted; 21% of the 92 students who were eligible). Engagement in conversations was unrelated to student age, sex, ethnicity, race, or class year (*p* > 0.35). Conversations were also unrelated to baseline importance of screening (*p* = 0.136), intentions (*p* = 0.244), and willingness to speak with family members (*p* = 0.240).

#### 3.4.1. Participants Who Engaged in Conversations

Of these participants, 10 (53%) reported speaking with family members about cancer screening since the baseline survey. Among participants who engaged in conversations, nine spoke with their mother (90%), none spoke with their father (0%), one spoke with grandparents (10%), and two spoke with other adults (20%). Two participants spoke with multiple family members (20%). With regard to the content of conversations, 9/10 (90%) spoke about breast cancer screening; 1/10 (10%) spoke about cervical cancer screening; 1/10 (10%) spoke about colorectal cancer screening, and 0/10 (0%) spoke about lung cancer screening.

Free response descriptions about how the conversations went were uniformly positive. The concept of *comfort* was raised by several students. Some participants mentioned they were nervous engaging in the conversations (“I felt semi comfortable because it was my mom but hard because bringing it up is always a little weird”; “I was a little uncomfortable at first discussing this but I know it’s an important topic and needed to be talked about”), but most described feeling generally at ease (“I felt very comfortable with this discussion and so did my mom;” “I felt very comfortable talking with my mom about this”). Several brought up the notion of *family member surprise* with discussions followed by appreciation (“My mom was surprised I brought this up, but she was happy I was taking responsibility;” “My mother was apprehensive as to why I was asking such a weird question. I explained that its important and I took a study survey in the fall on if your parents got cancer screenings. She then understood and appreciated me looking out for her health.”). Reports on family member screening activities varied. Some students mentioned their family members reported up-to-date screening, implying *limited potential efficacy* of discussion (“My mom indicated she had both [cervical and breast] done already and said it doesn’t hurt for me to get them done”. “She had then told me that she gets screened and makes sure to get checked up on regularly to make sure she doesn’t have cancer especially since some cancers run in our family”. “She stated that she has these appointments every year”). Two students reported that their family member immediately scheduled screening, implying *demonstrated impact* (“She got a mammogram immediately after”; “My mom actually booked one a few days after our conversation.”), while another reported agreement about importance but no action to date (“They agreed but haven’t gotten it yet.”). Reports on certainty regarding screening activities included discussion of *direct observation of results* (“I am sure both of my parents received screenings for cancer because they told me and showed the results”; “I am sure my mother received screening because she showed me the results of the examination.”), while others felt certain but offered no reasons for this perception (*perceived certainty*; e.g., “I’m positive my mom has had her screenings, however, I could not tell if my father had some screenings done or not.” “I know for a fact that a lot of them have to get screened.”). The concept of *need for additional information before discussions* was raised by two participants (“I possibly could have benefitted more from knowing the statistics of breast cancer.” “…for the most part I think it went really well. Maybe more knowledge on when and where to get screenings.”).

#### 3.4.2. Participants Who Did Not Engage in Conversations

Nine participants did not talk with family members about cancer screening. The most common reported reasons for not talking with family members about screening was forgetting about it (*n* = 7; 78%) and feeling too busy with other things (*n* = 7; 78%), while a smaller number of students reported feeling their parents/other adults were too busy (*n* = 3; 33%), or there was never the right time (*n* = 3; 33%). Only 3/9 (33%) participants felt that something might have made them engage in these conversations: two reported that a death or diagnosis would spur on conversations, and one felt that it would take a recommendation from their own doctor/provider.

## 4. Discussion

The current study sought to demonstrate the feasibility of an intervention using college students to increase cancer screening among their adult family members. Interval screening for cancer remains one of the most effective methods our healthcare system has for mitigating cancer-related negative outcomes [1,2], and results observed in this study highlight the potential for intergenerational interventions to complement existing cancer screening efforts [38,39,40]. We demonstrated that, as previously observed [30,31], college students are generally willing to discuss cancer screening with their family members, further emphasizing the acceptability of interventions in this area and population. This baseline willingness was not consistent across family members, as students reported greater willingness to speak with mothers than fathers or grandparents. Relatedly, students were generally more willing to discuss breast cancer screening than cervical, colorectal, or lung cancers. These independently replicated findings highlight the need for intergenerational interventions to place particular emphasis on broader discussion targets and topics.

The current study intentionally narrowed the targeted student population to only those who expressed high willingness to talk with family members and high individual intentions to receive cancer screenings as a way to maximize engagement, follow-up, and feedback. This approach was shown to be effective, as roughly ½ of students met criteria; 59% agreed to receive the intervention and accept a follow-up, and 35% completed follow-up. While this degree of interest in intervention participation was acceptable, subsequent intervention trials with a less tailored population will require greater efforts at encouraging engagement (e.g., theory-based messages targeting motivations, intentions, and/or perceived family member susceptibility to cancer [41,42,43,44]; increased monetary incentive for trial participation).

Among those who completed follow-up, student engagement in conversations mirrored the overall baseline willingness data: the majority of discussions were with mothers and focused on breast cancer. Although the frequency of cancer-related discussions between students and family members was encouraging in this feasibility trial (>50% of followed participants), intervention trials will require materials/information to maximize impact. Intergenerational interventions, like the one tested here, have the potential to directly impact more individuals than enrolled since a target participant can speak with multiple family members, but this was only observed twice in the current study. The low-intensity, information-based intervention provided in this feasibility study did not include any discussion of or encouragement for broad conversation topics and targets; future full trials will require these efforts to realize the potential impact of intergenerational interventions.

Nearly 50% of participants who completed follow-up reported that they did not talk with family members about cancer screening, with primary reasons being forgetting about it and feeling too busy. These data, while not surprising, provide important insights for future trials, as intervention materials should highlight to students the potential brevity of meaningful cancer screening discussions with family members. They also demonstrate the potential utility of booster materials, like theory-based motivational text reminders.

## 5. Limitations

There are several limitations to this study. First, as mentioned above, the sample used for intervention was selected to allow for the evaluation of operational feasibility and identify areas of need. As such, estimates of intervention engagement and outcomes are tenuous, requiring replication or disconfirmation in a larger trial. Second, although longitudinal methods were employed, little causal attribution can be made since this feasibility sample was small and no control (i.e., non-interventional) group was evaluated at follow-up. Subsequent work should make use of a larger sample and random assignment. Third, students self-reported on cancer screening conversations, such that there is potential for social desirability bias. Efforts to corroborate discussions through family member interviews would benefit future work. Fourth, we did not collect reasons for discussions with one family member (e.g., mother) and not with another (e.g., father).

## 6. Conclusions

This study illustrated the feasibility of using college students in a brief intervention to increase cancer screening behaviors among family members. An intervention utilizing lessons learned in this and previous studies [30,31] has a high likelihood of operational success.

## Figures and Tables

**Table 1:** Participant Characteristics.

	Mean	Standard Deviation	Frequency	Percentage
Age in Years	18.9	1.1		
Female Sex			126	67%
Race				
White			136	72%
Black			23	12%
Asian			10	5%
Native American			6	3%
Multi-racial			8	4%
Unknown/Refused			6	3%
Hispanic Ethnicity			30	16%
Class Status				
Freshmen			110	58%
Sophomore			47	25%
Junior			25	13%
Senior			7	4%
Presence of a Mother Figure			180	95%
Presence of a Father Figure			163	86%

## Data Availability

The data presented in this study are available on request from the corresponding author due to privacy concerns of participants associated with mixed-methods, small-sample research.
